# Evaluation of the Relationship Between Albuminuria and Triglyceride Glucose Index in Patients with Type 2 Diabetes Mellitus: A Retrospective Cross-Sectional Study

**DOI:** 10.3390/medicina61101803

**Published:** 2025-10-08

**Authors:** Ozgur Yilmaz, Osman Erinc

**Affiliations:** Department of Internal Medicine, Kanuni Sultan Suleyman Training and Research Hospital, Atakent Distr., Turgut Ozal Blvd., No: 46/1, Kucukcekmece, 34303 Istanbul, Turkey; doctorerinc@gmail.com

**Keywords:** insulin resistance, diabetic nephropathy, metabolic indices, renal function

## Abstract

*Background and Objectives*: Albuminuria is a key clinical marker for early detection of diabetic kidney disease (DKD) in individuals with type 2 diabetes mellitus (T2DM). The triglyceride-glucose (TyG) index, a simple surrogate of insulin resistance, has been increasingly investigated for its potential association with renal complications. This study aimed to evaluate the relationship between the TyG index and albuminuria in patients with T2DM and assess its clinical utility as an accessible metabolic marker reflecting early renal involvement. *Materials and Methods*: This retrospective cross-sectional study included 570 adult patients with confirmed T2DM who were followed at a tertiary internal medicine outpatient clinic between January and December 2024. Participants were classified as albuminuric or non-albuminuric based on spot urine albumin-to-creatinine ratio (ACR) values. Clinical and biochemical parameters were collected from medical records, and the TyG index was calculated as ln [fasting triglyceride (mg/dL) × fasting glucose (mg/dL)/2]. Logistic regression models were used to identify independent factors associated with albuminuria. ROC analysis was performed to evaluate the discriminatory accuracy of the TyG index. *Results*: The median TyG index was significantly higher in the albuminuric group compared to the non-albuminuric group (10.0 vs. 9.1; *p* < 0.001) and increased progressively with albuminuria severity (*p* < 0.001). In multivariate logistic regression analysis, elevated TyG index, hyperlipidemia, and reduced estimated glomerular filtration rate were independently associated with albuminuria. When evaluated as a continuous variable, the TyG index showed strong discriminatory ability (area under curve (AUC) = 0.949; 95% confidence interval (CI): 0.933–0.964). Using the optimal cut-off threshold of 9.6, the TyG index maintained high diagnostic performance (AUC = 0.870; 95% CI: 0.839–0.902; sensitivity 87.7%, specificity 86.3%). Subgroup analyses confirmed the robustness of this association across clinical and demographic variables. *Conclusions*: In this study, higher TyG index values were significantly associated with the presence and severity of albuminuria in individuals with T2DM. While causality cannot be inferred, the findings suggest that the TyG index may serve as a practical, cost-effective tool for identifying patients at increased risk for early diabetic kidney involvement. Prospective longitudinal studies are needed to confirm its predictive value and clinical applicability.

## 1. Introduction

Type 2 diabetes mellitus (T2DM) is a leading global health concern, affecting approximately 537 million adults as of 2023, with projections reaching 783 million by 2045 [1]. This growing prevalence, especially in developing regions, imposes a significant burden on healthcare systems [2], largely due to the microvascular and macrovascular complications associated with the disease. Diabetic kidney disease (DKD) is one of the most prevalent and costly complications of T2DM. Epidemiological studies report that approximately 30–40% of individuals with T2DM develop DKD during the course of their disease [3,4]. DKD is a leading cause of morbidity and mortality in diabetic patients and represents the most common etiology of end-stage renal disease (ESRD) worldwide [5]. In the United States, diabetes accounts for nearly 44% of patients initiating dialysis therapy, highlighting its clinical and economic burden on healthcare systems [6]. While these figures underline the importance of early identification and intervention in diabetic kidney disease to prevent progression to ESRD, similar trends have been reported globally. In Malaysia, for instance, diabetes contributes to approximately 49% of new end-stage renal disease (ESRD) cases requiring renal replacement therapy [7]. Moreover, a global analysis reported that the proportion of prevalent ESRD patients with diabetes increased from 19.0% in 2000 to 29.7% in 2015 worldwide, with particularly sharp increases observed in Asia and Latin America [8]. Together, these findings highlight the global dimension of diabetic kidney disease and the need for continued international attention to its prevention and management.

Albuminuria is a widely recognized early indicator of diabetic nephropathy, arising from structural disruption of the glomerular filtration barrier, including basement membrane alterations and podocyte injury, and plays a critical role in predicting the progression of renal damage [9,10,11]. Modern clinical and epidemiologic studies reinforce this: albuminuria remains one of the strongest prognostic markers for both renal and cardiovascular outcomes in patients with T2DM [12]. Importantly, microalbuminuria serves not only as a predictor of progression to macroalbuminuria and renal function decline but also correlates with heightened all-cause and cardiovascular mortality [11,12,13].

However, recent research has increasingly recognized that in T2DM, renal dysfunction may develop even in the absence of albuminuria, a phenomenon termed “non-albuminuric diabetic kidney disease” [14]. This emerging phenotype accounts for approximately 20–40% of DKD cases and challenges the traditional view that albuminuria is a prerequisite for diabetic renal decline. These observations underscore the heterogeneity in DKD pathogenesis and suggest that relying solely on albuminuria may overlook a significant subset of patients with progressive renal impairment [15]. A growing body of evidence suggests that insulin resistance (IR) plays a central and multifaceted role in the pathogenesis of DKD, extending well beyond hyperglycemia alone. It contributes to renal injury via endothelial dysfunction, enhanced oxidative stress, chronic low-grade inflammation, and activation of the renin-angiotensin-aldosterone system, ultimately promoting glomerular damage, tubulointerstitial fibrosis, and vascular injury even in the absence of albuminuria [16,17]. Multiple clinical studies have consistently demonstrated that insulin resistance (IR) is independently associated with both the presence and severity of albuminuria in individuals with type 2 diabetes mellitus. In a cross-sectional study using the gold-standard hyperinsulinemic-euglycemic clamp, lower insulin sensitivity was independently related to an increased risk of microalbuminuria [18]. Similarly, in a large cohort of patients with type 2 diabetes, higher HOMA-IR values were significantly associated with elevated urinary albumin excretion after adjustment for major confounders [19]. More recently, a nationwide cohort study from Korea confirmed that insulin resistance remained an independent risk factor for microalbuminuria, further reinforcing this association [20].

Given the heterogeneous nature of diabetic kidney disease and the limitations of albuminuria as a sole marker of renal dysfunction, recent research has increasingly focused on alternative metabolic indices that may better reflect underlying disease mechanisms. Among these, the triglyceride glucose (TyG) index has emerged as a practical, reliable, and independent surrogate marker of IR, with growing evidence linking it to renal and vascular complications in T2DM [21,22,23,24,25].

The TyG index, derived from fasting triglyceride and glucose measurements, has been widely validated as a reliable surrogate marker of insulin resistance in diverse populations and is easily applicable in routine clinical practice. Initial validation studies by Simental-Mendía et al. and Vasques et al. confirmed its strong correlation with clamp-derived IR and the Homeostasis Model Assessment of Insulin Resistance (HOMA-IR), respectively [26,27]. Moreover, evidence from large population-based cohorts indicates that the TyG index may provide superior diagnostic performance compared with HOMA-IR in identifying insulin resistance and predicting metabolic disorders, thereby reinforcing its clinical applicability [28,29]. Beyond its established role in metabolic syndrome, nonalcoholic fatty liver disease, and cardiovascular disease, TyG’s application to DKD has attracted growing attention [30,31]. Evidence from large-scale population-based studies across Asia and the United States demonstrates a consistent association between elevated TyG index levels and adverse renal outcomes, including albuminuria, chronic kidney disease and the development of ESRD [32,33,34]. Furthermore, TyG has shown superior or comparable diagnostic performance to other IR measures, including HOMA-IR, visceral adiposity index, and Lipid Accumulation Product, in predicting kidney outcomes among diabetic populations [35]. Together, these findings underscore the potential role of TyG as a metabolic biomarker linked to albuminuria and DKD, warranting further investigation.

Despite the growing body of research supporting the TyG index as a marker of IR, its relationship with diabetic kidney disease, particularly albuminuria, remains insufficiently explored. Existing studies have predominantly focused on cardiovascular and hepatic complications, with only limited cross-sectional data addressing renal outcomes. Moreover, the majority of available evidence derives from Asian populations, raising questions about the generalizability of these findings to other ethnic and geographic groups [31,36,37].

This study hypothesizes that the TyG index is independently associated with diabetic kidney disease and may serve as an early detection marker. The rationale lies in the need for a simple, inexpensive, and accessible biomarker to identify disease at an early stage and allow timely intervention. The objective was to examine the association between the TyG index and diabetic kidney disease in type 2 diabetes and to evaluate its predictive utility in clinical practice.

## 2. Materials and Methods

### 2.1. Study Design and Setting

This single-center, retrospective, cross-sectional study was conducted in the internal medicine outpatient clinic of a tertiary care hospital, which functions as a regional referral center for endocrine and metabolic diseases by providing advanced diagnostic and therapeutic services and receiving referrals from surrounding primary and secondary care facilities and included patients who presented between 1 January 2024 and 31 December 2024. Clinical and laboratory data of adult patients diagnosed with T2DM were retrospectively retrieved from the hospital’s electronic medical record system. All personal identifiers were anonymized prior to analysis to ensure patient confidentiality. As this was a retrospective analysis of anonymized data, the requirement for informed consent was waived by the ethics committee. This study was designed and structured in accordance with the STROBE (Strengthening the Reporting of Observational Studies in Epidemiology) guidelines.

### 2.2. Study Population

**Figure 1:**
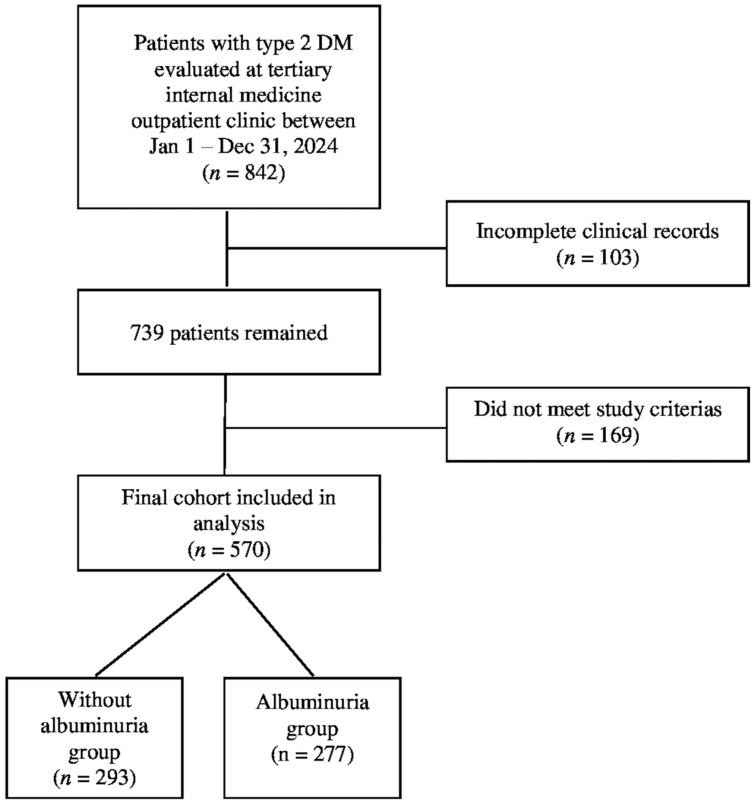
The determination of all participants.

A total of 842 adult patients diagnosed with T2DM were initially screened from the hospital’s electronic medical records. Following the application of the predefined inclusion and exclusion criteria, 272 patients were excluded due to missing essential laboratory data, a diagnosis of non-diabetic kidney diseases, or meeting other exclusion parameters. Consequently, 570 patients with complete clinical and laboratory information were included in the final analysis. The determination of all participants was summarized in Figure 1. The diagnosis of T2DM was confirmed according to the 2024 American Diabetes Association (ADA) guidelines.

The final study population was stratified into two groups based on urinary albumin excretion status. The first group, defined as the nonalbuminuric group, included patients without albuminuria, characterized by a spot urine albumin to creatinine ratio (ACR) of less than 30 mg/g. The second group, defined as the albuminuric group, consisted of patients with either microalbuminuria (ACR 30–300 mg/g) or macroalbuminuria (ACR > 300 mg/g), in accordance with the ADA and Kidney Disease: Improving Global Outcomes guidelines [38]. To accurately assess albuminuria status, the mean of at least two separate spot urine ACR measurements obtained during follow-up visits was used, thereby reducing the likelihood of misclassification due to transient clinical conditions.

### 2.3. Exclusion Criteria

Patients were excluded from the study if they met any of the following conditions: a diagnosis of type 1 diabetes mellitus; pregnancy or a history of gestational diabetes; the presence of nondiabetic kidney diseases such as glomerulonephritis, lupus nephritis, or polycystic kidney disease; chronic liver diseases; active malignancies; or ongoing immunosuppressive therapy, including corticosteroids and chemotherapy. Patients with advanced heart failure, a recent history of severe infections, or systemic inflammatory diseases (e.g., rheumatoid arthritis, systemic lupus erythematosus) were also excluded. In addition, patients with incomplete biochemical data or without at least two albumin-to-creatinine ratio measurements, as well as those presenting with acute clinical conditions known to cause transient albuminuria (such as urinary tract infections or febrile illnesses), were excluded to ensure accurate classification and data integrity.

### 2.4. Data Collection and Laboratory Measurements

All clinical and laboratory data were retrospectively retrieved from the hospital’s electronic medical records. Demographic characteristics including age, sex, and diabetes duration were recorded. Anthropometric data, such as height and weight, were used to calculate the body mass index (BMI) using the standard formula [weight (kg)/height (m)^2^]. All blood and urine samples were collected following an overnight fast of at least 8 h and processed in the hospital’s central biochemistry laboratory, which is accredited by national and international external quality assurance programs. Internal quality control was performed on a daily basis, and the laboratory regularly participated in external quality assessment schemes, with performance consistently within acceptable limits. All instruments were calibrated according to manufacturer instructions, and coefficients of variation for key assays (fasting glucose, triglycerides, HbA1c, creatinine, and urinary albumin) were maintained below 5%. Fasting blood glucose (FBG) and triglyceride levels were measured using an enzymatic colorimetric method on a Roche Cobas 8000 c702 analyzer (Roche Diagnostics, Mannheim, Germany). Glycated hemoglobin (HbA1c) was determined by high-performance liquid chromatography (HPLC) using the Tosoh G8 Analyzer (Tosoh Bioscience, Tokyo, Japan). Serum creatinine was measured using the kinetic Jaffe method on a Roche Cobas 8000 c702 analyzer (Roche Diagnostics, Mannheim, Germany), and the estimated glomerular filtration rate (eGFR) was calculated according to the Modification of Diet in Renal Disease equation. Urinary albumin concentration was measured using an immunoturbidimetric method on the Siemens ADVIA 1800 chemistry system (Siemens Healthcare Diagnostics, Erlangen, Germany), and urinary creatinine was measured using the Jaffe method on the same platform. The spot urine ACR was calculated accordingly. To ensure reliability and minimize the effect of transient factors, the mean of at least two separate ACR measurements obtained during routine outpatient follow-up visits was used for albuminuria classification.

### 2.5. Triglyceride Glucose Index Measurement and Calculation

The TyG index was calculated using the following formula: TyG index = ln [fasting triglyceride (mg/dL) × fasting plasma glucose (mg/dL)/2]. Laboratory measurements were conducted on the Roche Cobas 8000 c702 analyzer (Roche Diagnostics, Mannheim, Germany) using enzymatic colorimetric assays.

### 2.6. Study Variables

The dependent variable in this study was the presence of albuminuria, categorized according to urinary ACR thresholds (<30 mg/g for non-albuminuria, 30–300 mg/g for microalbuminuria, and >300 mg/g for macroalbuminuria). The primary independent variable was the TyG index. Additional independent variables included demographic characteristics (age, sex, duration of diabetes), metabolic parameters (fasting blood glucose, triglycerides, HbA1c, LDL-C, HDL-C, body mass index, hyperlipidemia), and clinical variables (serum creatinine, estimated glomerular filtration rate, and comorbid conditions).

### 2.7. Outcomes

The primary outcome of this study was the presence of albuminuria in patients with type 2 diabetes mellitus, classified by urine albumin-to-creatinine ratio (ACR) into non-albuminuric, microalbuminuric, and macroalbuminuric categories. The main objective was to assess whether the triglyceride-glucose (TyG) index is independently associated with albuminuria after adjustment for conventional metabolic and clinical risk factors. Secondary analyses included the relationship between TyG index values and the severity of albuminuria, as well as the discriminatory performance of the TyG index in identifying patients with albuminuria.

### 2.8. Statistical Analysis

All statistical analyses were performed using IBM SPSS Statistics for Windows, version 26.0 (IBM Corp., Armonk, NY, USA). As this was a retrospective cross-sectional analysis, no a priori sample size calculation was performed. However, a post-hoc power analysis indicated that the final cohort of 570 patients provided sufficient statistical power (>80% at α = 0.05) to detect significant associations between the TyG index and albuminuria. The normality of continuous variables was assessed using the Kolmogorov–Smirnov test. Continuous variables were expressed as means ± standard deviations (SD) or medians with interquartile ranges (IQR), as appropriate. Categorical variables were presented as frequencies and percentages. Comparisons between the albuminuric and non-albuminuric groups were performed using the independent samples t-test or Mann–Whitney U test for continuous variables and the chi-square (χ^2^) test for categorical variables. For comparisons across more than two groups, the Kruskal–Wallis test was applied, followed by Dunn’s post-hoc test with Bonferroni correction to identify pairwise differences. Binary logistic regression analyses were conducted to identify independent predictors of albuminuria. Variables with a *p*-value < 0.05 in univariate analysis and those with established clinical relevance were included in the multivariate logistic regression models. Results were reported as odds ratios (ORs) with 95% confidence intervals (CIs). Receiver operating characteristic (ROC) curve analysis was used to evaluate the discriminatory ability of the TyG index, with the area under the curve (AUC), optimal cut-off value (determined using the Youden index), sensitivity, and specificity being reported. Additionally, forest plots were generated to display the effect sizes and 95% CIs from subgroup analyses of clinically relevant variables. All statistical tests were two-tailed, and a *p*-value < 0.05 was considered statistically significant.

## 3. Results

A total of 570 patients with T2DM were included in the study and classified into two groups based on albuminuria status: 293 patients without albuminuria and 277 patients with albuminuria. Among the albuminuric group, 183 patients (66.1%) had microalbuminuria, and 94 patients (33.9%) had macroalbuminuria. The overall study population consisted of 297 females (52.1%) and 273 males (47.9%). No statistically significant differences were observed between the two groups in terms of age (*p* = 0.831) or sex distribution (*p* = 0.823).

All demographic and clinical characteristics of the study groups are summarized in Table 1.

The TyG index was higher in patients with albuminuria compared to those without albuminuria. The median TyG index value was 9.1 in the group without albuminuria, while the median value was 10.0 in the group with albuminuria. This difference between the two groups was statistically significant (*p* < 0.001). A detailed presentation of these findings, together with other laboratory parameters, is provided in Table 2.

In univariate logistic regression analysis, multiple metabolic and renal parameters were significantly associated with the presence of albuminuria. In the multivariate model, hyperlipidemia, decreased eGFR, and an increased TyG index remained as independent predictors of albuminuria (*p* < 0.05). Detailed results are presented in Table 3.

**Figure 2:**
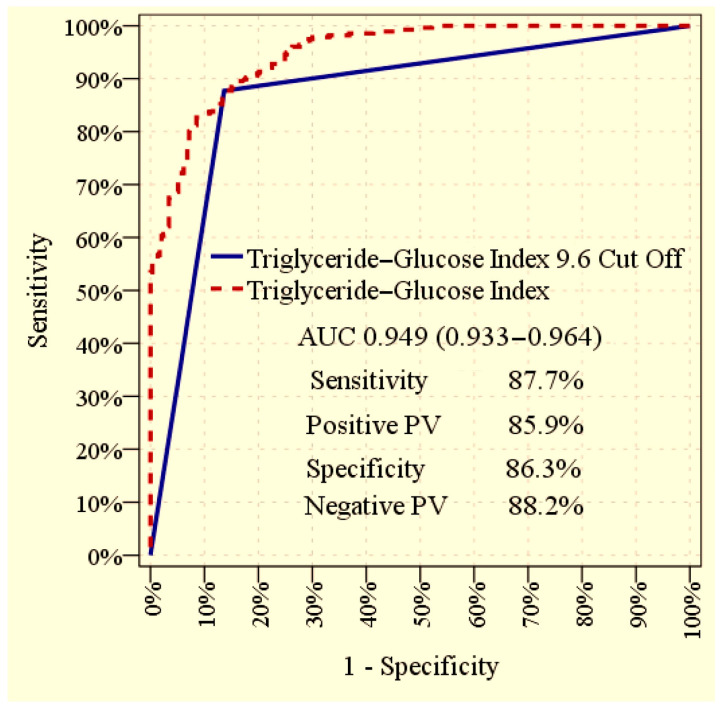
Receiver operating characteristic (ROC) curve illustrating the ability of the TyG index to discriminate between patients with and without albuminuria. The curves provide the overall diagnostic performance of the TyG index, both when used as a continuous variable and when applying the optimal cut-off value.

The ROC curve analysis revealed that the TyG index, when evaluated as a continuous variable, demonstrated an excellent ability to discriminate between patients with and without albuminuria, with an AUC of 0.949 (95% CI: 0.933–0.964; *p* < 0.001). Using the optimal cut-off value of 9.6 derived from the ROC curve, the TyG index maintained a high diagnostic performance, with an AUC of 0.870 (95% CI: 0.839–0.902; *p* < 0.001). At this cut-off point, the sensitivity was 87.7%, specificity was 86.3%, positive predictive value was 85.9%, and negative predictive value was 88.2% in differentiating albuminuric from non albuminuric patients. The graphical representation of the ROC analysis is displayed in Figure 2.

TyG index values increased progressively across albuminuria categories, with significant differences observed between all groups (*p* < 0.001). Detailed comparisons are presented in Table 4.

**Figure 3:**
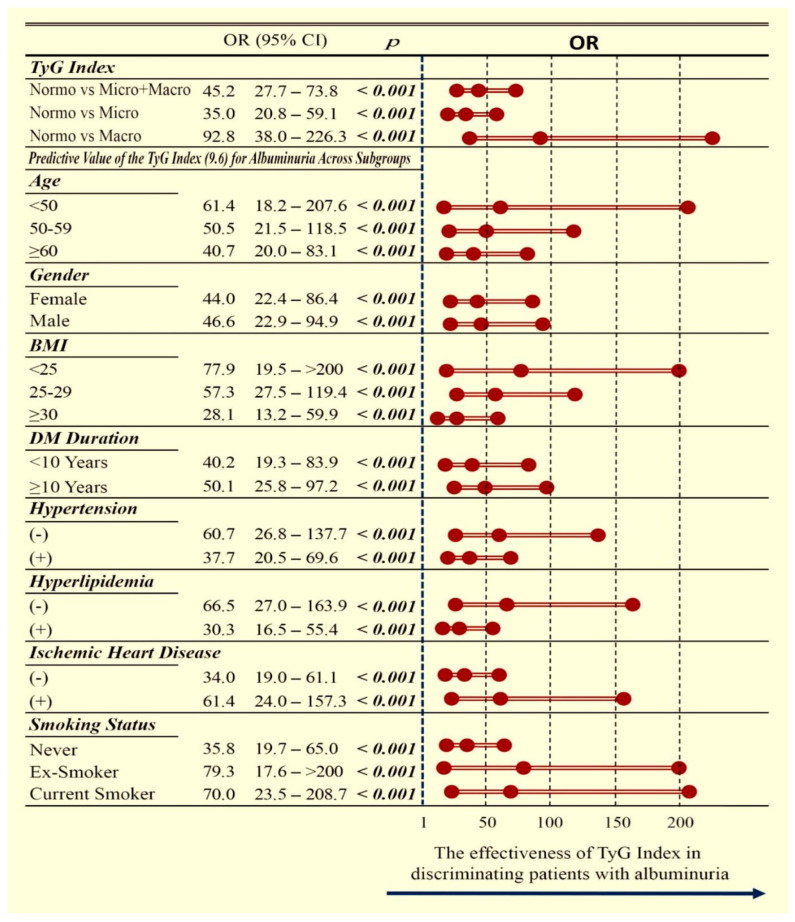
Forest plot showing the association between a TyG index above the cut-off value and albuminuria categories (normoalbuminuria, microalbuminuria, and macroalbuminuria). The plot highlights the direction and strength of the associations, allowing comparison across different categories and facilitating interpretation of the relative risk of renal involvement. OR: odds ratio; CI: confidence interval; TyG: triglyceride–glucose index; BMI: body mass index; DM: diabetes mellitus.

Multivariate subgroup analysis, illustrated in Figure 3, confirmed that a TyG index > 9.6 was strongly associated with the presence of albuminuria in the overall cohort and remained consistently predictive across microalbuminuria, macroalbuminuria, and all clinical subgroups. Detailed statistical outcomes are presented in Figure 3.

## 4. Discussion

The present study demonstrates that the TyG index is significantly and independently associated with early markers of DKD, including albuminuria and reduced eGFR, in individuals with type 2 diabetes mellitus. Higher TyG levels were observed in patients with albuminuria compared with those without, and a progressive increase in TyG values was noted across categories from normoalbuminuria to microalbuminuria and macroalbuminuria. Furthermore, hyperlipidemia was identified as an independent correlate, and a TyG cutoff value above 9.6 showed strong discriminative ability, with consistent accuracy across multiple clinical subgroups. Taken together, these findings support the potential role of the TyG index as a clinically relevant and cost-effective biomarker for the early stratification of renal risk in type 2 diabetes.

The present results corroborate and extend existing literature by using repeated ACR measurements to minimize misclassification and by demonstrating uniformity of effect through detailed subgroup analyses, thereby strengthening the evidence for the reliability of TyG as a marker of renal risk. A study conducted in China demonstrated a graded increase in the TyG index across albuminuria categories in over 1000 patients with T2DM [30], while another study from China showed that individuals in the highest TyG tertile were more than twice as likely to develop albuminuria compared with those in the lowest tertile [22]. In prospective cohort studies, elevated TyG was shown to predict incident DKD during follow-up [39], and its diagnostic performance was demonstrated to be superior to HOMA-IR [40]. The consistency of the findings with both cross-sectional and longitudinal data, including evidence from diverse populations such as a Turkish cohort [41], underscores the robustness and generalizability of the TyG-DKD association.

The biological plausibility of the TyG albuminuria association lies in its role as a surrogate of IR. IR promotes renal injury through endothelial dysfunction, oxidative stress, low-grade inflammation, and profibrotic signaling, which collectively accelerate glomerular injury and urinary albumin excretion [16,42,43,44]. Direct assessment of IR via clamp techniques is rarely feasible in clinical practice; thus, the TyG index provides a practical alternative. By reflecting systemic metabolic stress, TyG may capture both the hemodynamic and inflammatory components that contribute to the early pathogenesis of DKD.

This study further emphasizes the interplay between lipid abnormalities and renal injury. Patients with albuminuria exhibited higher triglycerides, LDLc, and total cholesterol, as well as lower HDLc. These findings are in line with evidence from Taiwan, where a lipid phenotype characterized by elevated triglycerides and apolipoprotein B conferred a more than threefold higher risk of albuminuria [45], and with data from China, where higher HDLc was reported to exert protective effects against microalbuminuria [36]. Together, these observations highlight that dyslipidemia not only accelerates atherosclerosis but also contributes directly to glomerular endothelial dysfunction, linking metabolic and vascular pathways of injury.

Inflammatory pathways also appear to mediate part of the observed associations. Although leukocyte count and CRP did not remain independent predictors in the multivariate models, their elevation among albuminuric patients suggests an underlying inflammatory milieu. Evidence from China has shown that leukocyte counts may partially mediate the TyG albuminuria association [37], and that TyG is correlated with high-sensitivity CRP [46]. These findings imply that systemic inflammation may act synergistically with metabolic stress to amplify renal damage, supporting the concept of DKD as a state of combined metabolic and inflammatory injury.

The identification of hyperlipidemia as an independent correlate in this cohort is noteworthy, as it reinforces the mechanistic link between metabolic dysregulation, DKD and cardiovascular pathology. Supporting this interplay, evidence from China demonstrated that the TyG index predicted cardiovascular risk in individuals with familial hypercholesterolemia [47], and also showed independent associations between TyG and heart failure with preserved ejection fraction among patients with coronary heart disease [48]. In line with these findings, the observation that coronary artery disease was more prevalent in patients with albuminuria highlights the convergent pathophysiology of vascular and renal complications in diabetes. Collectively, these parallels suggest that the TyG index may serve as a unifying biomarker reflecting systemic vascular vulnerability across multiple organ systems.

In this study, patients with albuminuria exhibited significantly lower eGFR, consistent with the concept that albuminuria frequently signals subsequent renal functional decline. This observation aligns with the findings from China, where the TyG index was shown to be inversely correlated with eGFR and positively associated with albuminuria, thereby highlighting its dual relationship with both functional impairment and structural injury in DKD. Together, these results strengthen the evidence supporting the TyG index as an informative biomarker of early renal dysfunction in T2DM [49].

In this cohort, multivariate logistic regression identified hyperlipidemia, reduced eGFR, and the TyG index as independent predictors of albuminuria. Notably, the TyG index exhibited a remarkably strong association, with an OR of 245.5 when modeled as a continuous variable. This effect size substantially exceeds previously reported estimates. For example, a study conducted in China described a threshold effect, whereby TyG values above approximately 9.05–9.09 were associated with an increased risk of biopsy-confirmed diabetic nephropathy [50]. Similarly, a prospective cohort study from China demonstrated that elevated TyG levels were independently associated with microalbuminuria and reduced eGFR, and that this predictive validity was maintained in longitudinal analyses [51]. Furthermore, a recent meta-analysis conducted in China confirmed the predictive value of TyG for chronic kidney disease, reinforcing its role as a reliable marker across diverse populations [52]. The significantly stronger association observed in this study may reflect differences in ethnic background, cohort characteristics, or the refined classification of albuminuria using the average of two ACR measurements.

The diagnostic capacity of the TyG index was further substantiated by ROC analysis, which yielded an AUC of 0.949 as a continuous variable. Using a cut-off of 9.6, TyG maintained robust discriminatory ability with an AUC of 0.870, demonstrating high sensitivity and specificity. Importantly, TyG values rose progressively across albuminuria categories from normoalbuminuria to microalbuminuria and macroalbuminuria, indicating a graded association with the severity of renal involvement. Subgroup analyses, illustrated by forest plots, confirmed that the predictive value of TyG was consistent across clinically relevant strata, including age, sex, BMI, diabetes duration, hypertension, hyperlipidemia, coronary artery disease and smoking status. The absence of effect modification across these categories suggests that the observed association is robust and may be generalizable. Collectively, these findings demonstrate the strong diagnostic accuracy of TyG and support its potential integration into risk stratification models for early diabetic kidney disease.

From a practical standpoint, the TyG cut-off of 9.6 should be viewed as a complement to—rather than a substitute for—standard DKD screening with HbA1c, ACR, and eGFR. Whereas HbA1c reflects long-term glycemic control and ACR/eGFR primarily captures established renal injury, TyG reflects upstream insulin-resistance–related risk and may therefore aid early risk stratification. In routine care, a TyG ≥ 9.6 could trigger repeat/confirmatory ACR testing, closer eGFR follow-up, and targeted metabolic optimization (lipids, glycemia, lifestyle), particularly in patients without known albuminuria. This approach may be useful in resource-limited settings because TyG is calculated from routine fasting lipids and glucose. At the same time, TyG should be interpreted alongside clinical context (fasting status, triglyceride variability, lipid-lowering therapy) and existing guideline tools; prospective studies are needed to confirm whether adding TyG to HbA1c, ACR, and eGFR improves DKD risk prediction and patient outcomes.

Despite these strengths, some limitations must be acknowledged. The retrospective and cross-sectional design limits causal inference, and the use of spot urine ACR, although averaged over two measurements, may not fully substitute for 24-h collections. Lack of systematically recorded data on lifestyle and behavioral factors (including dietary intake, physical activity, smoking intensity) and medication adherence represents another limitation, as these may influence both TyG index and albuminuria and could not be incorporated into the analysis. Furthermore, TyG was not directly compared with gold-standard measures of IR, and the single-center design may restrict external applicability. In addition, the cross-sectional nature of the study precludes assessment of longitudinal or predictive relationships. Although we observed a strong association between TyG index and albuminuria, the lack of longitudinal follow-up means we cannot establish whether elevated TyG index predicts the future development or progression of renal involvement. Prospective cohort studies are needed to confirm its prognostic value.

## 5. Conclusions

In conclusion, this study demonstrates that the TyG index is independently associated with albuminuria and reduced eGFR in type 2 diabetes, with strong discriminatory performance and robust associations across clinical subgroups. By capturing both metabolic and inflammatory dimensions of renal risk, TyG may serve as a practical, low-cost biomarker for early detection and stratification of DKD. Confirmation in longitudinal and multicenter cohorts will be essential to establish its role in clinical practice and to determine whether incorporating TyG into routine risk assessment can improve outcomes for patients with diabetes.

## Figures and Tables

**Table 1:** Comparison of demographic, anthropometric and clinical characteristics between patients with and without albuminuria.

	Without Albuminuria (*n* = 293)	With Albuminuria (*n* = 277)	*p*
Age, years	59 (52–65)	59 (52–66)	0.832
Gender, n (%)			
- Female	154 (52.6)	143 (51.6)	0.823
- Male	139 (47.4)	134 (48.4)	
Weight, kg	76 (70–85)	78 (71–87)	0.073
Body mass index, kg/m^2^	28.7 (26–31)	28.9 (26–31)	0.870
Systolic blood pressure, mmHg	130 (120–140)	130 (120–140)	0.225
Diastolic blood pressure, mmHg	85 (75–90)	85 (75–90)	0.433
Diabetes duration, years	10 (6–15)	11 (8–15)	0.176
Comorbid diseases, n (%)			
- Hypertension	177 (60.4)	165 (59.6)	0.837
- Hyperlipidemia	125 (42.7)	213 (76.9)	<0.001
- Ischemic heart disease	58 (19.8)	135 (48.7)	<0.001
Smoking status, n (%)			
- Never	186 (63.5)	169 (61)	0.774
- Current smoker	70 (23.9)	68 (24.5)	
- Ex-smoker	37 (12.6)	40 (14.4)	
Medication, n (%)			
- Oral antidiabetic drugs (OAD)	185 (63.1)	170 (61.4)	0.663
- Insulin therapy only	33 (11.3)	37 (13.4)	0.446
- OAD + Insulin	71 (24.2)	68 (24.5)	0.930
- Antihypertensive drug	156 (53.2)	150 (54.2)	0.828
- Lipid-lowering drug	125 (42.7)	213 (76.9)	<0.001

OAD: oral antidiabetic drugs. Values are expressed as median (IQR) or n (%). *p* values were calculated using the Mann–Whitney U test for continuous variables and chi-square (χ^2^) test for categorical variables.

**Table 2:** Comparison of TyG index and relevant laboratory parameters between groups.

Parameter	Without Albuminuria (*n* = 293)	With Albuminuria (*n* = 277)	*p*
Triglyceride–glucose index	9.1 (8.7–9.4)	10 (9.7–10.4)	<0.001
Fasting blood glucose, mg/dL	142 (111–177)	207 (175–265)	<0.001
Hemoglobin A1c, %	7.9 (6.8–9.3)	9 (7.8–10)	<0.001
Urea, mg/dL	30.3 (25–37)	34 (26–41)	0.001
Creatinine, mg/dL	0.79 (0.7–0.9)	0.95 (0.7–1.2)	<0.001
eGFR, mL/min/1.73 m^2^	101 (96–105)	75 (57–90)	<0.001
Uric acid, mg/dL	4.5 (3.7–5.5)	4.8 (4–6.4)	0.014
Aspartate aminotransferase, U/L	16 (13–20)	16 (13–20)	0.684
Alanine aminotransferase, U/L	17 (13–22)	17 (13–21)	0.418
Total cholesterol, mg/dL	185 (157–213)	196 (166–231)	0.011
Triglyceride, mg/dL	120 (94–157)	217 (186–275)	<0.001
HDL-c, mg/dL	45 (38–52)	43 (36–49)	0.009
LDL-c, mg/dL	108 (81–138)	116 (90–146)	*0.031*
C-reactive protein, mg/L	2.5 (1–6)	3.5 (1.4–8)	0.031
Leukocyte, 10^3^/mm^3^	7.9 (6.7–9.8)	8.3 (7.1–10)	0.035
UACR, mg/g	11.2 (5.9–20.3)	109.7 (50–454)	<0.001

eGFR: estimated glomerular filtration rate; HDL-c: high-density lipoprotein cholesterol; LDL-c: low-density lipoprotein cholesterol; UACR: urine albumin to creatinine ratio. Values are expressed as median (IQR). *p* values were calculated using the Mann–Whitney U test.

**Table 3:** Univariate and multivariate logistic regression analyses of factors associated with albuminuria.

Variable	Univariate Model					Multivariate Model				
	OR	95% CI			*p*	OR	95% CI			*p*
Hyperlipidemia	4.473	3.112	–	6.430	<0.001	2.628	1.240	–	5.569	0.012
Ischemic heart disease	3.852	2.656	–	5.586	<0.001					
Lipid-lowering drugs	0.224	0.156	–	0.321	<0.001					
Glucose, mg/dL	1.020	1.016	–	1.024	<0.001					
Hemoglobin A1c, %	1.311	1.190	–	1.443	<0.001					
Urea, mg/dL	1.020	1.007	–	1.032	0.002					
Creatinine, mg/dL	63.28	25.22	–	158.74	<0.001					
eGFR, mL/min/1.73 m^2^	0.858	0.834	–	0.882	<0.001	0.852	0.815	–	0.891	<0.001
Total cholesterol mg/dL	1.006	1.002	–	1.009	0.001					
Triglyceride, mg/dL	1.031	1.026	–	1.037	<0.001					
HDL-c, mg/dL	0.982	0.966	–	0.997	0.019					
LDL-c, mg/dL	1.006	1.001	–	1.010	0.010					
Triglyceride–glucose index	351.09	126.03	–	978.01	<0.001	245.5	68.2	–	883.5	<0.001

OR: odds ratio; CI: confidence interval; eGFR: estimated glomerular filtration rate; HDL-c: high-density lipoprotein cholesterol; LDL-c: low-density lipoprotein cholesterol; TyG: triglyceride–glucose index. ORs were derived from univariate and multivariate logistic regression analyses.

**Table 4:** Comparison of TyG index values across albuminuria categories.

	Normo-Albuminuria (*n* = 293)	Micro-Albuminuria (*n* = 183)	Macro-Albuminuria (*n* = 94)	*p*
TyG Index	9.1 ^2,3^ (8.7–9.4)	9.9 ^3^ (9.7–10.2)	10.2 (9.9–10.6)	<0.001

Data are presented as median [interquartile range] as appropriate. Comparisons among the three groups were performed using the Kruskal–Wallis test, followed by post hoc pairwise comparisons with Dunn’s method. ^2^ *p* < 0.05 vs. microalbuminuria group; ^3^ *p* < 0.05 vs. macroalbuminuria group. TyG: triglyceride–glucose index.

## Data Availability

The data that support the findings of this study are available from the corresponding author upon reasonable request.

## Abbreviations

TyG	triglyceride glucose index
T2DM	type 2 diabetes mellitus
DKD	diabetic kidney disease
ACR	albumin-to-creatinine ratio
ESRD	end-stage renal disease
IR	insulin resistance
HOMA-IR	homeostasis model assessment of insulin resistance
ADA	American diabetes association
BMI	body mass index
FBG	fasting blood glucose
HbA1c	glycated hemoglobin
eGFR	estimated glomerular filtration rate
HDLc	high-density lipoprotein cholesterol
LDLc	low-density lipoprotein cholesterol
ROC	receiver operating characteristic
AUC	area under curve
PV	predictive value
CI	confidence interval
OR	odds ratio
SD	standard deviation
IQR	interquartile range
